# Clinical Efficacy of Preoperative and Intraoperative Intravitreal Ranibizumab as Adjuvant Therapy of Ahmed Glaucoma Valve Implantation Combined with Vitrectomy in the Management of Neovascular Glaucoma with Diabetic Vitreous Hemorrhage

**DOI:** 10.3390/jpm14010018

**Published:** 2023-12-22

**Authors:** Shuang Gao, Zhongjing Lin, Yisheng Zhong, Xi Shen

**Affiliations:** 1 Department of Ophthalmology, Ruijin Hospital, Affiliated Shanghai Jiaotong University School of Medicine, Shanghai 200025, China; 2 Department of Ophthalmology, Renji Hospital, Affiliated Shanghai Jiaotong University School of Medicine, Shanghai 200127, China

**Keywords:** neovascular glaucoma, proliferative diabetic retinopathy, vitrectomy, Ahmed glaucoma valve implantation, ranibizumab

## Abstract

Neovascular glaucoma (NVG) secondary to proliferative diabetic retinopathy (PDR) is a devastating ocular disease with poor prognosis. Intravitreal ranibizumab injection (IVR) has been used as adjuvant therapy of surgical interventions preoperatively or intraoperatively. This study aimed to determine the efficacy and safety of combined IVR as adjuvant therapy in treating NVG with vitreous hemorrhage (VH) in PDR. A total of 39 NVG patients with VH (39 eyes) received IVR 3 to 5 days before surgery, and then they were assigned to either pars plana vitrectomy (PPV) + Ahmed glaucoma valve (AGV) implantation (Group 1, *n* = 22) or PPV + AGV implantation + intraoperative IVR (Group 2, *n* = 17). Patients were followed up for at least 9 months. Intraocular pressure (IOP), anti-glaucoma medications, best corrected visual acuity (BCVA), surgical success rates and postoperative complications were compared. Results showed that IOP decreased promptly after surgery and was notably maintained at a mid-term follow-up in both groups, and no significant differences were observed (all *p* > 0.05). Additional intraoperative IVR significantly reduced postoperative recurrent VH and iris neovascularization (*p* = 0.047, *p* = 0.025, respectively). There was no remarkable difference in postoperative anti-glaucoma medications, BCVA and complications between two groups (all *p* > 0.05). In conclusion, preoperative and intraoperative IVR as adjuvant therapy of AGV implantation combined with PPV could be a safe and effective treatment for NVG with VH in PDR. An additional intraoperative anti-VEGF injection could significantly reduce postoperative VH and iris neovascularization.

## 1. Introduction

Neovascular glaucoma (NVG), an intractable and blinding disease, is difficult to manage and always leads to disastrous vision loss [1]. It results from the pathological neovascularization of the anterior eye segment, which interferes with the normal drainage of aqueous humor [2]. The most common ocular diseases responsible for the progression of NVG are ischemic retinopathies, such as retinal vein occlusion and proliferative diabetic retinopathy (PDR). Nowadays, an increasing diabetic population promotes the higher prevalence of diabetic NVG [3]. It is reported that diabetes accounts for over 30% of NVG cases [4], while the incidence of iris neovascularization was as high as 65% in PDR [5]. As an advanced manifestation of PDR, NVG is regarded as the terminal diabetic ocular complication and closely associated with poor prognosis [6]. In order to avoid severe visual impairment in these PDR patients, the priority is to prevent its development of NVG through an appropriate and timely therapeutic strategy. According to European Glaucoma Society Guidelines, early detection and intervention of retinal ischemia is the most critical and essential management, which could minimize the development of subsequent neovascularization [7]. However, the optimal treatment for NVG secondary to PDR is a real challenge for clinical ophthalmologists. It usually requires both medication and surgery to control the PDR and sustained elevated intraocular pressure (IOP) [8]. The approaches to treat NVG include anti-glaucoma and anti-vascular endothelial growth factor (VEGF) therapy, pan-retinal photocoagulation (PRP) and surgical interventions. Trabeculectomy with mitomycin C or 5-FU is not an ideal operation choice for these complicated NVG patients. Based on our previous retrospective study, we found that Ahmed glaucoma valve (AGV) implantation was an effective and safe procedure that enabled vision preservation and successful IOP control in NVG patients who have undergone 23-gauge pars plana vitrectomy (PPV) previously for treating PDR [9]. We also found that AGV implantation combined with 23-gauge vitrectomy for PDR patients with medically uncontrolled NVG might be a safe alternative [10]. However, there are still many limitations due to their retrospective nature. A previous study has demonstrated that a comprehensive therapeutic strategy for NVG, including anti-VEGF therapy and anti-glaucoma surgery, could preserve visual function and control IOP [11]. The latest Cochrane review showed that anti-VEGF agents, as an adjunct to conventional treatment for NVG, could help reduce intraocular pressure (IOP) in the short term, but there is no evidence that long-term effectiveness of anti-VEGF drugs still exists [12]. Based on an observational study [13], both severe PDR and lack of postoperative intravitreal ranibizumab injection (IVR) were significant predictors of NVG for PDR patients after vitrectomy. Ranibizumab, a recombinant humanized IgG1 kappa isotype monoclonal antibody fragment directed against human VEGF-A, is one of the anti-VEGF agents used in daily clinical practice. Postoperative intravitreal anti-VEGF injection was identified as a protective factor for vision recovery [14]. Considering that increased vitreous VEGF level was regarded as a predictor factor in the development of PDR after vitrectomy [15], we attempt to optimize the therapeutic strategy for those PDR patients with NVG who also have indications for vitrectomy. Therefore, this prospective study aims to investigate whether another injection of anti-VEGF agent at the end of the surgery could improve the prognosis of PDR patients with NVG who have undergone vitrectomy and AGV implantation after preoperative anti-VEGF treatment.

## 2. Materials and Methods

This study was conducted in the department of Ophthalmology, Ruijin Hospital, affiliated to Shanghai Jiaotong University School of Medicine, between December 2021 and December 2022. With a thorough understanding of this study, all the enrolled patients signed an informed consent before the treatment. This study was registered at ClinicalTrials.gov (No. NCT05156021) accessed on 14 October 2021.

### 2.1. Study Population

This prospective study recruited patients from the department of ophthalmology in Ruijin Hospital. Enrolled PDR patients should meet the criteria as follows: complicated with NVG, including neovascularization in iris and/or anterior chamber angle and secondary elevated IOP (>22 mm Hg) which was not medically controlled, and requiring vitrectomy (including unabsorbed vitreous hemorrhage (VH), dense vitreous opacity resulted from previous hemorrhage and fibrovascular proliferation). The exclusion criteria were shown as follows: (1) coexistent ocular disease that may interfere with visual outcome; (2) rhegmatogenous and/or tractional retinal detachment; (3) NVG due to any other reasons except PDR (such as branch or central retinal vein occlusion, ocular ischemic syndrome); (4) previous history of intraocular surgery and anti-VEGF therapy; (5) severe external ocular infection; (6) other uncontrolled systemic diseases (such as hypertension, renal disease, and abnormal coagulation-associated blood disease); (7) irreversible blindness with no light perception. Only one eye of each patient was involved in this study. The preoperative preparations of these patients included ensuring that their fasting blood glucose level was lower than 8 mmol/L and postprandial blood glucose level was lower than 10 mmol/L for three days before surgery. All patients received preoperative antibiotic eye drops and anti-glaucoma eye drops (maximum dosage).

### 2.2. Surgical Procedures

PDR patients participating in this study all received one intravitreal injection of 0.5 mg ranibizumab (Lucentis, Novartis, Basel, Switzerland) 3 to 5 days before the surgery. After that, they were separated into two groups randomly. Both Group 1 and Group 2 received the AGV implantation and vitrectomy, but Group 2 received another IVR at the end of surgery. AGV implantation was performed by glaucoma specialist (Zhong YS), and vitrectomy was performed by vitreoretinal specialist (Shen X) for all patients. The surgical process was conducted as previously described [10]. After retrobulbar and topical anesthesia, the superotemporal conjunctiva was incised to make a fornix-based flap between rectus muscles. Soaked with 25 mg/mL 5-FU, a piece of cotton was put in this flap for 5 min, then a thorough rinse was conducted. After checking the tube patency, the plate of AGV (FP7; New World Medical, Inc., Rancho Cucamonga, CA, USA) was carefully fixed onto the sclera using 5–0 nylon sutures, with anterior edge posterior to the limbus for approximately 8–9 mm. After that, a 5 × 5 mm^2^ half-thickness limbal-based scleral flap was prepared. Subsequently, 23-gauge three-port PPV was performed. Endolaser photocoagulation was applied when necessary. The drainage tube was then inserted into the anterior chamber, with 2–3 mm tube in anterior chamber where the bevel faced upwards. The sclera flap was sutured by 10–0 nylon sutures. The conjunctiva was sutured with 8–0 vicryl sutures. After the operation, topical glucocorticoids and antibiotics were used. Eyeball massage or anti-glaucomatous medications were administered when required based on postoperative IOP changes. The follow-up period for all patients was at least 9 months.

### 2.3. Data Collection

Baseline characteristics of these patients were collected preoperatively, including age, gender, systemic condition (hypertension, HbA1c at time of surgery, duration of diabetes), previous PRP, lens status (phakic or pseudophakic), follow-up period, baseline best-corrected visual acuity (BCVA) and intraocular pressure. BCVA was assessed with logMAR BCVA, where Snellen visual acuity was converted to logMAR equivalents to facilitate further statistical analysis. Visual acuity of no light perception, light perception, hand motion and counting finger were assigned as 3.0, 2.6, 2.3 and 1.85 logMAR, respectively. After the treatment, postoperative IOP and anti-glaucoma medications of the two study groups were recorded at 1 week, 1 month, 3 months, 6 months and 9 months. Throughout the postoperative follow-up period, the intraoperative tamponade (balanced salt solution, air, silicone oil) and postoperative complications of these patients were also recorded, including the application of postoperative anti-VEGF injection, postoperative PRP, recurrent vitreous hemorrhage, recurrent retinal detachment, recurrent iris neovascularization and final BCVA.

### 2.4. Statistical Analysis

The Statistical Package for Social Sciences software version 20.0 (SPSS Inc, Chicago, IL, USA) was used for the statistical analysis. Quantitative data were expressed as mean ± standard deviation. The differences between the two groups were calculated using unpaired *t*-test or Fisher exact test, where appropriate. The rate of complete success (postoperative IOP between 6 and 21 mmHg without medications) and qualified success (postoperative IOP between 6 and 21 mmHg with or without medications) was also documented. *p* value < 0.05 was defined as statistical significance.

## 3. Results

A total of 39 eyes of 39 NVG patients with VH secondary to severe PDR were finally enrolled in this study. After IVR treatment, 22 eyes underwent PPV + AGV implantation (Group 1) and 17 underwent PPV + AGV implantation + intraoperative IVR (Group 2). The detailed clinical characteristics are summarized in Table 1. The mean age at surgery was 48.0 ± 14.6 years old in Group 1 and 45.5 ± 12.3 years old in Group 2 (*p* = 0.570). Similarly, there were no statistically significant differences in gender distribution, the portion of systemic hypertension, HbA1c levels and duration of diabetes (all *p* > 0.05). The portion with previous PRP in Group 1 and Group 2 was 40.9% and 35.3%, respectively, and no significant difference was established between the two groups (*p* = 0.721). The mean follow-up was 11.9 ± 2.3 months (range, 9–17 months) in Group 1 and 12.4 ± 2.5 months (range, 9–17 months) in Group 2 (*p* = 0.569). The baseline BCVA improved from 1.98 ± 0.38 to 1.46 ± 0.68 in Group 1, whereas changed from 1.96 ± 0.36 to 1.27 ± 0.57 in Group 2. The mean BCVA change showed no significant difference between the two groups (*p* = 0.428).

**Figure 1:**
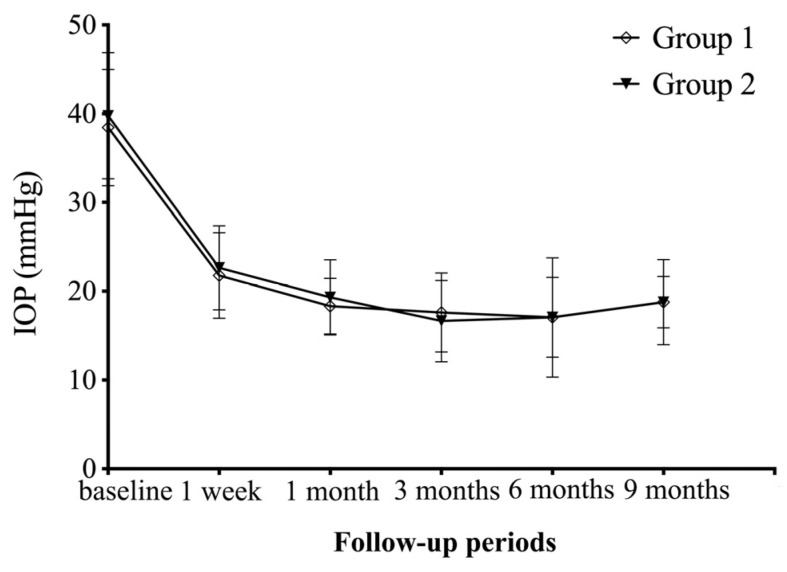
Graph showing IOP changes from baseline to 9 months after surgery.

Details of IOP, BCVA and anti-glaucoma medications at each follow-up visit are listed in Table 2. The mean preoperative IOP was 38.5 ± 6.6 mmHg in Group 1 and 39.8 ± 7.1 mmHg in Group 2 (*p* = 0.555). From the 1st week to the 6th month after operation, the IOP of the two groups showed a decreased trend, and there was no significant difference between the two groups at each time point (all *p* > 0.05) (Figure 1). Maximal topical anti-glaucoma medications were used on all eyes before the surgery, and postoperative medication use for both groups decreased at each of the time points as compared to preoperative amounts. However, there was no difference between the two groups (all *p* > 0.05). With respect to the postoperative surgical success rates, the complete success of IOP control was similar between the two groups (all *p* > 0.05). The qualified success rates of Group 1 at 3, 6 and 9 months were 68.18%, 77.27% and 63.64%, respectively, while Group 2 was found to achieve slightly higher portions (82.35%, 82.35%, 70.59%), but the differences did not reach significant levels (all *p* > 0.05) (Table 3).

Postoperative complications are reported in Table 4. The portions of postoperative anti-VEGF and PRP showed no significant differences between the two groups (*p* = 0.921, *p* = 0.648, respectively). The incidence of recurrent retinal detachment was similar between the two groups (*p* = 0.883). The portions of recurrent VH and iris neovascularization in Group 2 were significantly lower (*p* = 0.047, *p* = 0.025, respectively). During the whole follow-up periods, no intraoperative or postoperative sight-threatening complications occurred in any of the patients, such as expulsive hemorrhage, hypotony (IOP < 5 mmHg), bleb leak, choroidal detachment, AGV tube obstruction or endophthalmitis. Additionally, all patients maintained good control of blood glucose levels, and no patient developed systemic complications.

## 4. Discussion

With the growing morbidities of diabetes, the prevalence of NVG secondary to PDR demonstrates an increasing tendency. NVG with VH in PDR is a devastating ocular disease that may result in the recommendation of eyeball enucleation [16]. Such patients typically do not seek treatment until the vision of the affected eye becomes very poor and they suffer from unbearable pain. Currently, academics have not reached consensus about the optimal management strategy, due to significant paucity of large-scale and well-constructed randomized controlled trials in the management of NVG [17]. Further work is encouraged to determine the suitable time point for medication implementation, such as anti-VEGF therapy. In our current study, preoperative and intraoperative IVR combined with PPV and AGV implantation led to rapid regression of iris neovascularization, facilitated IOP control and improved BCVA with less postoperative complications in NVG cases.

The combination therapy provides several advantages in NVG patients with VH in PDR. AGV implantation is commonly regarded as an optimal surgical option for the management of NVG and provides satisfactory outcomes in terms of IOP control. Vitrectomy removes dense vitreous hemorrhage and fibrovascular proliferation, which clears the vitreous cavity and improves the postoperative visual acuity. PPV also removed the vitreous gel which might affect the retinal oxygenation, thus promoting the fast resolution of macular edema. In addition, PPV could facilitate the implementation of PRP in the operation. Moreover, PPV and PRP could significantly inhibit the expression of VEGF, thus reducing or preventing postoperative recurrent neovascularization. Therefore, simultaneous surgery of PPV and AGV implantation appears to an efficacious treatment for NVG with VH in PDR patients [10]. Bernal-Morales et al. [18] reviewed 51 NVG patients treated with PPV, PRP and AGV implantation. The cumulative success rates were as high as 74.4% at 12 months and 71.4% at 24 months, suggesting the combined surgery could achieve effective and sustained long-term postoperative IOP control.

When diabetic retinopathy progresses to PDR, VEGF is regarded as the most potent proangiogenic factor that plays an important role in the presence of pathological retinal neovascularization. As VEGF has a direct effect on the angiogenic cascade and fibroblast activity, intravitreal injection of anti-VEGF agents has been proposed as an adjuvant therapeutic option in the treatment of NVG [19,20]. Intravitreal injections of VEGF inhibitors have been reported to result in a significant decrease in the concentration of VEGF in the aqueous humor and vitreous body. Preoperative administration of anti-VEGF agents has been shown to induce rapid regression of fragile new vessels in the iris and the anterior chamber angle, thus effectively reducing the occurrence of intraoperative hyphema and increasing surgical success after AGV implantation. On the other hand, it could facilitate the removal of vitreous hemorrhage, thus ensuring the safety of the subsequent multiple surgical procedures [12,21]. The preoperative use of IVR as adjuvant therapy in the management of NVG combined with VH has been described in previous reports. Wang et al. [22] allocated 18 NVG eyes with VH to IVR plus PPV and AGV implantation, and IVR was administered 3–7 days prior to the combined surgery. A significant reduction in IOP compared to baseline was achieved (49.83 ± 8.66 mmHg vs. 16.92 ± 2.75 mmHg), and surgical success rates maintained at 71.3% at 12-month visit.

Although preoperative injections of anti-VEGF drugs have been widely employed to promote the rapid regression of neovascularization and improve the visual prognosis, the benefit effect of IVR pretreatment may be transient, recurrent VH after successful vitrectomy is not uncommon. Early postoperative VH is often related with injured retinal vessels, residual fibrinous clots or early rapid recurrent formation of neovascularization. In the late stage after the surgery, neovascularization remained after vitrectomy may contribute to the increased VEGF and inflammatory cytokines in the aqueous humor, which in turn leads to the recurrence of VH and NVG [23]. Tang et al. [24] compared the efficacy of preoperative IVR plus AGV implantation with AGV implantation alone in NVG eyes. They found there was no significant difference in IOP control at 12-month follow-up and concluded that a single preoperative IVR had no significant effect on the long-term surgical outcome of AGV implantation in NVG cases. Therefore, the efficacy of another intraoperative IVR at the end of the surgery was investigated in this clinical trial.

Mean IOP decreased promptly after surgery and was notably maintained at a mid-term follow-up in these two groups. Although the majority of NVG patients with VH are effectively treated with the combined surgical interventions, some patients still necessitate additional PRP and anti-VEGF to control persistent retinal ischemia or elevated IOP. Considering the qualified success rates were slightly higher in Group 2, additional intraoperative IVR might contribute to the lowering of IOP more efficiently. There was no remarkable difference in postoperative visual acuity between the two groups. The potential explanation may be that all these NVG cases had developed to the advanced stage and the underlying retinal disease was severe. Moreover, additional intraoperative IVR significantly reduced the incidence of postoperative recurrent VH and iris neovascularization. Similarly, Ahn et al. [25] demonstrated that intraoperative administration of intravitreal bevacizumab seemed to be a better option to decrease postoperative VH after vitrectomy. Another issue worthy to be considered is that the clearance of IVR is more rapid in vitrectomized eyes, thus the benefit effect of anti-VEGF agents would be very short [26], necessitating repeated injections during the long-term follow-up periods. Thus, researchers have postulated the necessity for novel drug delivery systems that would maintain long-term therapeutic effects without the need for repeated injections. To sum up, NVG with simultaneous VH in PDR generally carries a poor prognosis; however, such miserable outcomes have a chance to decline due to the combined surgical interventions with additional intraoperative IVR.

Although repeated intravitreal anti-VEGF injections could improve prognosis in most cases, it would also bring about significant economic burden to family and society that could not be ignored. In order to reduce this public health burden, researchers try to develop sustained-release therapeutic strategies for anti-VEGF agents that could allow longer duration of action with fewer required reinjections. Sustained-release anti-VEGF drugs are manufactured as more potent and slowly dissolved agents, which could suppress pathological neovascularization over an extended period. The non-degradable implants, the Port Delivery System with ranibizumab, has currently been tested in phase III clinical trial [27]. However, such non-degradable implants need to be removed through secondary surgery. Considering this risk is unavoidable, more researchers have put their emphasis on those biodegradable slowly released drugs, for which the releasing rate can be adjusted through changing its molecular weight and composition [28]. Polylactic-Co-Glycolic Acid (PLGA) [29], hydrogel-based drug delivery systems [30], liposomes [31] and light-responsive nanoparticles [32] were all biodegradable materials that might be noninvasive, more efficient sustained-release strategies for the application of anti-VEGF agents. However, investigations of these carriers are mostly in the basic research stages and still have a long way to go. In addition, many patients exhibited insensitivity to anti-VEGF treatments. Although many potential targets for treating pathological neovascularization have been discovered, anti-VEGF drugs were irreplaceable in clinical use at the current stage. Therefore, adequate PRP is crucial for these patients to avoid recurrence of neovascularization. It is reported that a delay in application of PRP beyond 31 days was significantly associated with worse visual prognosis than those treated earlier [33]. Based on our previous findings [34], there is insufficient evidence to recommend anti-VEGF therapy as an alternative to PRP. A consensus has been reached that anti-VEGF treatment alone might be insufficient for preventing new vessel progression, especially in those with severe and rapidly progressing NVG. PRP is able to eliminate the production and release of vaso-proliferative factors through destroying capillary nonperfusion areas in the retina, thereby effectively reducing the oxygen demand and inhibiting pathological retinal angiogenesis. If initial PRP is inadequate, new vessel recurrence would happen when the effects of anti-VEGF drugs disappear. Supplemental PRP could promote faster regression of neovascularization and facilitate earlier stable IOP control. Although PRP would contribute to visual disturbance gradually, extensive and sufficient PRP must be performed even though temporary new vessel regression is achieved after application of anti-VEGF agents. Last but not least, individualized therapy relying on baseline condition, level of compliance and economic status is the vital part in enhancing therapeutic effect and optimizing vision outcome.

There are certain limitations to disclose in our current study. The number of enrolled patients was quite small. The duration of follow-up varied but was relatively short. Another limitation worthy to note was that this was a single-center study. Further explorations are warranted to extend our present observations, with larger samples and multiple medical centers enrolled. Even so, we provided an alternative surgical approach in the management of medically uncontrolled NVG and VH in PDR patients.

## 5. Conclusions

Preoperative and intraoperative IVR as adjuvant therapy of AGV implantation combined with PPV could be a safe and effective treatment for NVG with VH in PDR. An additional intraoperative anti-VEGF injection could significantly reduce postoperative VH and iris neovascularization.

## Figures and Tables

**Table 1:** The basic clinical characteristics of the study population.

	Group 1	Group 2	*p* Value
Number of eyes	22	17	
Age (years)	48.0 ± 14.6	45.5 ± 12.3	0.570
Gender (male/female)	10/12	7/10	0.789
Systemic profile			
Hypertension	15 (68.2%)	11 (64.7%)	0.819
HbA1c at time of surgery	8.6 ± 2.2	9.1 ± 2.2	0.521
Duration of diabetes (years)	14.0 ± 4.8	12.4 ± 5.1	0.325
Previous PRP	9 (40.9%)	6 (35.3%)	0.721
Lens status			0.966
Phakic	18 (81.82%)	14 (82.35%)	
Pseudophakic	4 (18.18%)	3 (17.65%)	
Follow-up (months)	11.9 ± 2.3	12.4 ± 2.5	0.569
Baseline BCVA	1.98 ± 0.42	1.96 ± 0.36	0.894

Categorical variables were expressed as *n* (%). PRP, pan-retinal photocoagulation; BCVA, best-corrected visual acuity.

**Table 2:** Postoperative IOP and anti-glaucoma medications of the study groups throughout follow-up periods.

Follow-Up Periods	IOP			BCVA			Number of Anti-Glaucoma Medications		
	Group 1	Group 2	*p* Value	Group 1	Group 2	*p* Value	Group 1	Group 2	*p* Value
1 week	21.8 ± 4.8	22.6 ± 4.7	0.576	2.03 ± 0.40	1.96 ± 0.30	0.567	1.4 ± 1.1	1.1 ± 1.0	0.370
1 month	18.3 ± 3.2	19.3 ± 4.3	0.416	1.83 ± 0.44	1.67 ± 0.52	0.330	1.1 ± 0.9	0.8 ± 1.0	0.381
3 months	17.6 ± 4.5	16.6 ± 4.6	0.521	1.75 ± 0.51	1.62 ± 0.50	0.410	0.9 ± 1.0	1.0 ± 0.9	0.763
6 months	17.0 ± 6.7	17.1 ± 4.5	0.994	1.68 ± 0.61	1.58 ± 0.54	0.569	1.0 ± 1.0	0.8 ± 0.8	0.432
9 months	18.8 ± 4.8	18.8 ± 2.9	0.995	1.48 ± 0.67	1.29 ± 0.56	0.363	1.0 ± 1.0	0.9 ± 0.8	0.687

**Table 3:** Postoperative surgical success rates of the study groups throughout follow-up periods.

Follow-Up Periods	Complete Success			Qualified Success		
	Group 1	Group 2	*p* Value	Group 1	Group 2	*p* Value
1 week	7 (31.82%)	6 (35.29%)	>0.999	10 (45.45%)	6 (35.29%)	0.744
1 months	7 (31.82%)	8 (47.06%)	0.508	15 (68.18%)	12 (70.59%)	>0.999
3 months	10 (45.45%)	5 (29.41%)	0.343	15 (68.18%)	14 (82.35%)	0.464
6 months	9 (40.91%)	8 (47.06%)	0.754	17 (77.27%)	14 (82.35%)	>0.999
9 months	9 (40.91%)	6 (35.29%)	0.753	14 (63.64%)	12 (70.59%)	0.740

**Table 4:** The intraoperative tamponade and postoperative complications of the study population.

	Group 1	Group 2	*p* Value
Intraoperative tamponade			0.709
BSS	7 (31.8%)	4 (23.5%)	
Air	5 (22.7%)	3 (17.6%)	
Silicone oil	10 (45.5%)	10 (58.8%)	
Postoperative anti-VEGF	10 (45.5%)	8 (47.1%)	0.921
Postoperative PRP	8 (36.4%)	5 (29.4%)	0.648
Recurrent VH	7 (31.82%)	1 (2.56%)	0.047
Recurrent RD	6 (27.3%)	5 (29.4%)	0.883
Recurrent iris neovascularization	8 (36.36%)	1 (2.56%)	0.025
Final BCVA	1.46 ± 0.68	1.27 ± 0.57	0.361
Change in BCVA (Final–Baseline)	−0.52 ± 0.76	−0.69 ± 0.52	0.428

Categorical variables were expressed as *n* (%). BSS, balanced salt solution; PRP, panretinal photocoagulation; anti-VEGF, anti-vascular endothelial growth factor; VH, vitreous hemorrhage; RD, retinal detachment; BCVA, best-corrected visual acuity.

## Data Availability

The datasets used and/or analyzed during the current study are included in this article.

## References

[1] Hayreh S.S. (2007). Neovascular glaucoma. Prog. Retin. Eye Res..

[2] Simha A., Aziz K., Braganza A., Abraham L., Samuel P., Lindsley K.B. (2020). Anti-vascular endothelial growth factor for neovascular glaucoma. Cochrane Database Syst. Rev..

[3] Tang Y., Shi Y., Fan Z. (2023). The mechanism and therapeutic strategies for neovascular glaucoma secondary to diabetic retinopathy. Front. Endocrinol..

[4] Kalogeropoulos D., Moussa G., Sung V.C., Pappa C., Kalogeropoulos C. (2023). Neovascular Glaucoma: An Update. Klin. Monbl Augenheilkd..

[5] Senthil S., Dada T., Das T., Kaushik S., Puthuran G.V., Philip R., Rani P., Rao H., Singla S., Vijaya L. (2021). Neovascular glaucoma—A review. Indian. J. Ophthalmol..

[6] Shazly T.A., Latina M.A. (2009). Neovascular glaucoma: Etiology, diagnosis and prognosis. Semin. Ophthalmol..

[7] European Glaucoma Society (2017). European Glaucoma society terminology and guidelines for glaucoma, 4th edition—Chapter 2: Classification and terminologySupported by the EGS foundation: Part 1: Foreword; introduction; glossary; chapter 2 classification and terminology. Br. J. Ophthalmol..

[8] Sánchez-Tabernero S., Juberías J.R., Artells N., Crespo-Millas S., Meneses C., Muñoz-Moreno M.F., Manzanas L., López Gálvez M.I. (2019). Management and Systemic Implications of Diabetic Neovascular Glaucoma. Ophthalmic Res..

[9] Cheng Y., Liu X.H., Shen X., Zhong Y.S. (2013). Ahmed valve implantation for neovascular glaucoma after 23-gauge vitrectomy in eyes with proliferative diabetic retinopathy. Int. J. Ophthalmol..

[10] Lin Z.J., Chen Z.H., Huang S.Y., Sun J., Shen X., Zhong Y.S. (2020). Clinical efficacy of Ahmed glaucoma valve implantation combined with 23-gauge vitrectomy for medically uncontrolled neovascular glaucoma with proliferative diabetic retinopathy. Int. J. Ophthalmol..

[11] Sun Y., Liang Y., Zhou P., Wu H., Hou X., Ren Z., Li X., Zhao M. (2016). Anti-VEGF treatment is the key strategy for neovascular glaucoma management in the short term. BMC Ophthalmol..

[12] Rittiphairoj T., Roberti G., Michelessi M. (2023). Anti-vascular endothelial growth factor for neovascular glaucoma. Cochrane Database Syst. Rev..

[13] Liang X., Zhang Y., Li Y.P., Huang W.R., Wang J.X., Li X. (2019). Frequency and Risk Factors for Neovascular Glaucoma After Vitrectomy in Eyes with Diabetic Retinopathy: An Observational Study. Diabetes Ther..

[14] Wang S., Liu Y., Du Y., Bao H., Zhu J., Liu X. (2023). Influencing factors of low vision 2 years after vitrectomy for proliferative diabetic retinopathy: An observational study. BMC Ophthalmol..

[15] Wang J., Chen S., Jiang F., You C., Mao C., Yu J., Han J., Zhang Z., Yan H. (2014). Vitreous and plasma VEGF levels as predictive factors in the progression of proliferative diabetic retinopathy after vitrectomy. PLoS ONE.

[16] Shchomak Z., Cordeiro Sousa D., Leal I., Abegão Pinto L. (2019). Surgical treatment of neovascular glaucoma: A systematic review and meta-analysis. Graefes Arch. Clin. Exp. Ophthalmol..

[17] Ramji S., Nagi G., Ansari A.S., Kailani O. (2023). A systematic review and meta-analysis of randomised controlled trials in the management of neovascular glaucoma: Absence of consensus and variability in practice. Graefes Arch. Clin. Exp. Ophthalmol..

[18] Bernal-Morales C., Dotti-Boada M., Olate-Perez A., Navarro-Angulo M.J., Pelegrín L., Figueras-Roca M. (2021). Simultaneous pars plana vitrectomy, panretinal photocoagulation, cryotherapy, and Ahmed valve implantation for neovascular glaucoma. Int. J. Ophthalmol..

[19] Zhou M., Xu X., Zhang X., Sun X. (2016). Clinical outcomes of Ahmed glaucoma valve implantation with or without intravitreal bevacizumab pretreatment for neovascular glaucoma: A systematic review and meta-analysis. J. Glaucoma.

[20] Lin P., Zhao Q., He J., Fan W., He W., Lai M. (2022). Comparisons of the short-term effectiveness and safety of surgical treatment for neovascular glaucoma: A systematic review and network meta-analysis. BMJ Open.

[21] Pei M., Zhao X., Wan G. (2023). A systematic review and meta-analysis of clinical outcomes of small gauge vitrectomy with or without intravitreal antivascular endothelial growth factor agents pretreatment for proliferative diabetic retinopathy. Ophthalmic Res..

[22] Wang M.H., Li Q.M., Dong H.T., Dong S.Q., Li Y., Zheng C.Y. (2017). Ahmed valves vs trabeculectomy combined with pans plana vitrectomy for neovascular glaucoma with vitreous hemorrhage. Eur. J. Ophthalmol..

[23] Gao S., Lin Z., Chen Y., Xu J., Zhang Q., Chen J., Shen X. (2020). Intravitreal conbercept injection as an adjuvant in vitrectomy with silicone oil infusion for severe proliferative diabetic retinopathy. J. Ocul. Pharmacol. Ther..

[24] Tang M., Fu Y., Wang Y., Zheng Z., Fan Y., Sun X., Xu X. (2016). Efficacy of intravitreal ranibizumab combined with Ahmed glaucoma valve implantation for the treatment of neovascular glaucoma. BMC Ophthalmol..

[25] Ahn J., Woo S.J., Chung H., Park K.H. (2011). The effect of adjunctive intravitreal bevacizumab for preventing postvitrectomy hemorrhage in proliferative diabetic retinopathy. Ophthalmology.

[26] Edington M., Connolly J., Chong N.V. (2017). Pharmacokinetics of intravitreal anti-VEGF drugs in vitrectomized versus non-vitrectomized eyes. Expert. Opin. Drug Metab. Toxicol..

[27] Holekamp N.M., Campochiaro P.A., Chang M.A., Miller D., Pieramici D., Adamis A.P., Brittain C., Evans E., Kaufman D., Maass K.F. (2022). Archway Randomized Phase 3 Trial of the Port Delivery System with Ranibizumab for Neovascular Age-Related Macular Degeneration. Ophthalmology.

[28] Xu M., Fan R., Fan X., Shao Y., Li X. (2022). Progress and Challenges of Anti-VEGF Agents and Their Sustained-Release Strategies for Retinal Angiogenesis. Drug Des. Devel Ther..

[29] Tanetsugu Y., Tagami T., Terukina T., Ogawa T., Ohta M., Ozeki T. (2017). Development of a Sustainable Release System for a Ranibizumab Biosimilar Using Poly(lactic-co-glycolic acid) Biodegradable Polymer-Based Microparticles as a Platform. Biol. Pharm. Bull..

[30] Yu Y., Lin X., Wang Q., He M., Chau Y. (2019). Long-term therapeutic effect in nonhuman primate eye from a single injection of anti-VEGF controlled release hydrogel. Bioeng. Transl. Med..

[31] Abrishami M., Zarei-Ghanavati S., Soroush D., Rouhbakhsh M., Jaafari M.R., Malaekeh-Nikouei B. (2009). Preparation, characterization, and in vivo evaluation of nanoliposomes-encapsulated bevacizumab (avastin) for intravitreal administration. Retina.

[32] Huu V.A., Luo J., Zhu J., Zhu J., Patel S., Boone A., Mahmoud E., McFearin C., Olejniczak J., Lux C.d.G. (2015). Light-responsive nanoparticle depot to control release of a small molecule angiogenesis inhibitor in the posterior segment of the eye. J. Control Release.

[33] Ohlhausen M., Payne C., Greenlee T., Chen A.X., Conti T., Singh R.P. (2021). Impact and Characterization of Delayed Pan-Retinal Photocoagulation in Proliferative Diabetic Retinopathy. Am. J. Ophthalmol..

[34] Gao S., Lin Z., Shen X. (2020). Anti-Vascular Endothelial Growth Factor Therapy as an Alternative or Adjunct to Pan-Retinal Photocoagulation in Treating Proliferative Diabetic Retinopathy: Meta-Analysis of Randomized Trials. Front. Pharmacol..

